# Effects of Film Thickness of ALD-Deposited Al_2_O_3_, ZrO_2_ and HfO_2_ Nano-Layers on the Corrosion Resistance of Ti(N,O)-Coated Stainless Steel

**DOI:** 10.3390/ma16052007

**Published:** 2023-02-28

**Authors:** Mihaela Dinu, Kaiying Wang, Emile S. Massima Mouele, Anca C. Parau, Alina Vladescu (Dragomir), Xinhua Liang, Viorel Braic, Leslie Felicia Petrik, Mariana Braic

**Affiliations:** 1 National Institute of Research and Development for Optoelectronics INOE 2000, 409 Atomistilor St., 077125 Magurele, Romania; 2 Department of Energy, Environmental & Chemical Engineering, Washington University in St. Louis, St. Louis, MO 63130, USA; 3 Department of Separation Science, School of Engineering Science, Lappeenranta-Lahti University of Technology LUT, Yliopistonkatu 34, FI-53850 Lappeenranta, Finland; 4 Physical Materials Science and Composite Materials Centre, Research School of Chemistry & Applied Biomedical Sciences, National Research Tomsk Polytechnic University, Lenin Avenue 43, Tomsk 634050, Russia; 5 Department of Chemistry, Environmental and Nano Sciences, University of the Western Cape, Robert Sobukwe Road, Bellville 7535, South Africa

**Keywords:** atomic layer deposition, oxides, oxynitride, corrosion resistance

## Abstract

The goal of this stydy was to explore the potential of the enhanced corrosion resistance of Ti(N,O) cathodic arc evaporation-coated 304L stainless steel using oxide nano-layers deposited by atomic layer deposition (ALD). In this study, we deposited Al_2_O_3_, ZrO_2_, and HfO_2_ nanolayers of two different thicknesses by ALD onto Ti(N,O)-coated 304L stainless steel surfaces. XRD, EDS, SEM, surface profilometry, and voltammetry investigations of the anticorrosion properties of the coated samples are reported. The amorphous oxide nanolayers homogeneously deposited on the sample surfaces exhibited lower roughness after corrosion attack compared to the Ti(N,O)-coated stainless steel. The best corrosion resistance was obtained for the thickest oxide layers. All samples coated with thicker oxide nanolayers augmented the corrosion resistance of the Ti(N,O)-coated stainless steel in a saline, acidic, and oxidising environment (0.9% NaCl + 6% H_2_O_2_, pH = 4), which is of interest for building corrosion-resistant housings for advanced oxidation systems such as cavitation and plasma-related electrochemical dielectric barrier discharge for breaking down persistent organic pollutants in water.

## 1. Introduction

The exposure of stainless steel (SS) to harsh environments may result in corrosion that further limits its performance and durability. It is therefore necessary to develop an effective method to prevent corrosion. For decades, thin films have been studied because of their exceptional physical and chemical features including excellent thermal stability, low friction coefficient, and good wear resistance [1]. These properties have been improved by means of multilayer deposition onto selected supports, although their application depends on cost and practicality. Physical vapor deposition (PVP), such as the reactive arc evaporation process, has long been used as an adequate technique to develop monolayer and multilayer coatings. Titanium nitride (TiN) films obtained by PVD are claimed to show restricted corrosion protection ability due to their fundamental permeability. The increase in film thickness has been identified as a practical approach in order to enhance corrosion resistance [2]. Moreover, the use of bi- and multi-layered coatings containing two layers with distinct composition was productively used in engineering applications such as the automotive, aircraft, and tool industries [1]. The superior features are the result of the presence of interfaces that ensure crack deflection, thus providing enhanced ductility and decreased stress levels, resulting in higher adhesion to the bulk substrate. In corrosive environments, the interfaces block the access of liquid to the bulk support, providing superior corrosion resistance.

Subramanian et al. investigated the corrosion protection ability of the titanium nitride coatings TiN, TiON, and TiAlN for biomedical applications [3] and their results showed that the CP–Ti/TiAlN coating exhibited higher corrosion performance. Ti(N,O) coatings represent a valuable option due to their superior mechanical and tribological properties. The quite recent studies on titanium oxynitride films explored their dependency on the N/O ratio, which allow their use as solar selective absorbers [4], biocompatible materials [5,6], plasmonic material for nano-photonics [7], or photocatalytic coatings [8,9], to name a few. We previously reported on the improved corrosion resistance of cathodic acr deposited TiN coating in 0.10 M NaCl + 1.96 M H_2_O_2_ solution with low oxygen concentration (about 7.3 at.%), compared to TiN coatings obtained using the same deposition method [10]. Even if TiN coatings are known to be corrosion resistant, Esaka et al. demonstrated using absorption and X-ray photoelectron spectroscopies that the oxidation of TiN films is ascribable to the formation of Nx–Ti–Oy-like structures, permitting the diffusion of oxygen from the corrosive solution. Thus, a decrease in the corrosion resistance of TiN compared to CrN is observed, in which Cr_2_O_3_ is immediately formed at the coating–solution interface, without the formation of Nx–Cr–Oy-like structures [11]. In addition, Wang et al. demonstrated that in an O_2_ environment, TiN coating, unlike stainless steel, showed lower corrosion resistance than in a H_2_ environment [12]. 

Our aim is to use corrosion-resistant, coated SS as a material for building corrosion-resistant housings for advanced oxidation systems such as cavitation and plasma-related electrochemical dielectric barrier discharge for breaking down persistent organic pollutants in water [13,14,15,16], because normal SS corrodes very quickly in these systems. 

Attempting to further improve the corrosion resistance of Ti(N,O) coatings with a low oxygen content, we apply a second layer of ultrathin oxide coating due to the known good chemical and mechanical stability of oxide films [17]. Because TiN and Ti(N,O) grown by CAE present a compressive stress, we chose to use a deposition method that may produce a very thin, continuous and conformal coating to avoid delamination of the two layers. These dense and conformal films are expected to diminish the pinhole and droplet density specific for the CAE-deposited coatings, enabling sealing even with thin coatings of nanometre thicknesses [18]. Even though various approaches have been used to prepare thin films, it is still challenging to obtain a smooth and conformal dense film. Previous investigations claimed that ALD is an advanced method to engineer films of good quality at the nanoscale [19,20]. Atomic layer deposition (ALD) is a self-limiting reaction that involves the use of a precursor and oxidant followed by a flow of inert gas [21,22]. The common advantages of ALD include the control of film thickness, minor structural pinholes, low defect density, and good film uniformity [23,24]. These unique properties justify the extensive application of ALD for film coatings over the past decades [25,26,27,28,29]. Fedel and Deflorian performed atomic layer-deposited Al_2_O_3_ films on AISI 316L stainless steel [30]. The corrosion resistance of Al_2_O_3_ deposits were evaluated by potentiodynamic measurements and electrochemical impedance spectroscopy (EIS) in 0.2 M NaCl saline environment for up to 1000 h of non-stop immersion. Their outcomes showed that the corrosion current density of the bare support (10^−6^ A/cm^2^) declined proportionally with the increase in the deposition layers Al_2_O_3_–500 (10^−8^ A/cm^2^) and Al_2_O_3_–1000 (10^−9^ A/cm^2^), respectively. Their results suggested that the addition of Al_2_O_3_ layers greatly shielded the bare AISI 316L stainless steel against corrosive aggression. Belén Díaz et al. engineered ultra-thin (5 to 50 nm) films of aluminium and tantalum oxides by ALD at two deposition temperatures (250 °C and 160 °C) onto a 316L stainless steel support [31]. The corrosion protection behaviour of the coatings was investigated by linear scan voltammetry (LSV) and electrochemical impedance spectroscopy (EIS) in 0.8 M NaCl saline medium. The results indicated that the current density decreased by up to four orders of magnitude with an increase of coating thickness from 5 to 50 nm. In both cases, thicker coatings (50 nm) of Al_2_O_3_ and Ta_2_O_5_ exhibited the greatest corrosion resistance at 250 °C, with Al_2_O_3_ having superior shielding properties. These examples show that ALD is an ideal method because it is capable of depositing ultrathin, conformal films with sub-nanometre thickness control [32]. ALD has been widely used in anti-corrosion applications without diminishing the desirable functions of the support [18,32,33,34]. Generally, inorganic coatings, especially ceramics, show good insulating, tribological, and corrosion resistance properties in aggressive media. Al_2_O_3_ is the most frequently studied ALD thin film for corrosion protection, because it has been shown to nucleate well on metals, giving rise to low porosity that prevents the solution from accessing the metal [32,35,36]. ZrO_2_ reveals excellent properties such as high strength, high fracture toughness, excellent wear resistance, high hardness, and excellent chemical resistance [17,26,37,38,39]. HfO_2_ is a highly resistive material; its dielectric constant is several times higher than conventional silica gate dielectrics. The Pourbaix diagram shows the formation of a stable passive oxide on hafnium over the entire potential pH range. Due to these features, thin layers of HfO_2_ are applied for anticorrosion protection [17,39].

In this study, we coated Al_2_O_3_, ZrO_2_, and HfO_2_ thin films of various thicknesses on stainless steel support surfaces using ALD to investigate their anticorrosion performance. We theorise that Al_2_O_3_, ZrO_2_ and HfO_2_ prepared using ALD could improve the corrosion protection properties of the Ti(N,O) coating that we previously developed using CAE onto stainless steel supports [10]. Based on our information, the deposition on stainless steel supports of different layers obtained by CAE and then by ALD has not been reported to date. The rationale of this approach is based on the attempt to combine the chemical stability of oxide films obtained by ALD, which perfectly enrobe the support, and the high hardness and adhesion of the coatings synthetised by CAE. Furthermore, we used an accelerated corrosion test using saline, acidic, and oxidising solution. To our knowledge, there are few examples of tests carried out in such corrosive solutions [40,41,42] on ALD-coated supports. The corrosion protection behaviour of the fabricated coatings was assessed by potentiodynamic polarisation tests in a simulated corrosive environment (0.9% NaCl + 6% H_2_O_2_, pH = 4). The 0.9% NaCl solution is often used as an initial baseline test for understanding the fundamental response of the metal or coating [43]. The addition of H_2_O_2_ mimics the solutions used in an advanced oxidation system, and that is the reason for using the aggressive test solution 0.9%NaCl + 6% H_2_O_2_. Noteworthy is that the chloride ion is one of the important driving forces of corrosion for steels, but it consumes hydroxyl radicals. To prevent this, we added 6% H_2_O_2_, because at a low pH of the solution and a H_2_O_2_ concentration higher than 10 mg/L, the consumption of the hydroxyl radicals by chlorine is hindered, permitting the presence of H_2_O_2_ for a few hours in the saline solution [44]. The effect of the number of ALD cycles on the ceramic film thickness, growth mechanism, and corrosion resistance were also investigated.

## 2. Materials and Methods

All coatings were deposited on 304L stainless steel, abbreviated as SS. The composition of 304 SS rods (provided by Bibus Metals AG, Fehraltorf, Switzerland), from which the discs were machined, is (wt.%): Fe = 70.974%, Cr = 17.742%, Ni = 8.526%, Mn = 1.23%, Mo = 0.585%, Cu = 0.536%, Si = 0.206%, Co = 0.16%, P = 0.021%, S = 0.014%, and C = 0.006%.

### 2.1. Coating Deposition

According to a previous study [10], PVD-deposited Ti(O,N) showed the highest protection efficiency for SS against NaCl corrosive attack. 

To summarise, the Ti(N,O) coatings were deposited on SS discs (20 mm diameter and 2 mm thickness) using a Ti cathode (99.5% purity, Cathay Advanced Materials Ltd., Guangzhou, China) by reactive CAE in a mixture of nitrogen and oxygen. We chose 304L SS because compared to other types of stainless steels, it has a low carbon content, such that it presents a higher corrosion resistance. Moreover, it is non-magnetic after annealing, which might be of interest for certain applications. 

The discs were first polished with abrasive paper (80 to 800 mesh size) and then repeatedly polished with a 0.5 μm diamond suspension to reach a roughness (Ra) of about 13 nm. The discs were cleaned using an ultrasonic bath in acetone, isopropyl alcohol, and distilled water, and then were flushed with dried nitrogen. Two Si coupons of 10 × 10 mm were used for the measurement of the coatings, covered with 2 mm Si band firmly attached to the Si coupons, so as to provide a non-coated area with a sharp edge. After being introduced into the deposition chamber, on a rotating holder that ensured the uniformity of the coating, a residual pressure of 5 × 10^−4^ Pa was attained. Then, the discs were bombarded by 1 keV Ar^+^ at 10^−2^ Pa for final cleaning. The Ti(N,O) deposition parameters were as follows: total gas pressure during deposition = 8 × 10^−2^ Pa; N_2_ flow rate = 60 sccm; O_2_ flow rate = 17 sccm; arc current on Ti cathode = 90 A; support bias voltage = −200 V; support temperature = 200 °C. All of the 21 SS discs were coated with Ti(N,O) in one run due to the large diameter of the deposition chamber (80 cm).

**Figure 1:**
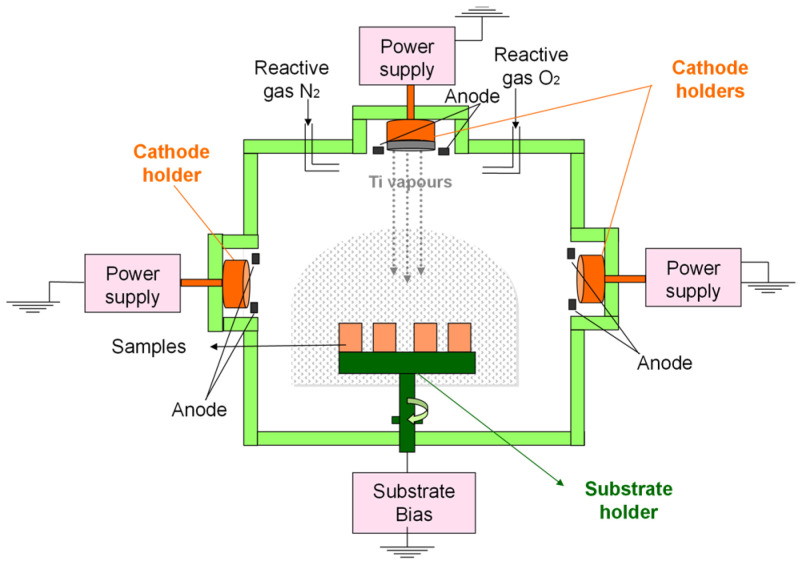
Setup for the cathodic arc deposition.

Figure 1 presents the setup for the cathodic arc deposition.

In the present work, ALD was employed to further inhibit the corrosion of the prepared SS/Ti(N,O) using Al_2_O_3_, ZrO_2_, and HfO_2_ layer deposition. The precursors were trimethylaluminum (TMA, Sigma Aldrich, St. Louis, MO, USA) and deionised (DI) water for Al_2_O_3_ ALD, tetrakis(dimethylamido)zirconium (TDMAZ, Sigma Aldrich) and 30 wt.% hydrogen peroxide (Sigma Aldrich) in water for ZrO_2_ ALD, and tetrakis(diethylamido)hafnium (TDEAH, Sigma Aldrich) and 30 wt.% hydrogen peroxide in water for HfO_2_ ALD.

For the Al_2_O_3_ ALD, 40 and 80 cycles of Al_2_O_3_ ALD (40c- and 80c-Al_2_O_3_) were applied; for the ZrO_2_ ALD, 45 and 90 cycles of ZrO_2_ ALD were applied; and for the HfO_2_ ALD, 55 and 110 cycles of HfO_2_ ALD were applied. Each type of oxide coating with two thickness was deposited on three SS/Ti(N,O) samples.

In a typical run, the pieces of SS/Ti(N,O) were degassed at 150 °C overnight under nitrogen (N_2_) atmosphere before ALD. All of the ALD processes were carried out at 200 °C. All precursor feed lines were kept above 120 °C to prevent the condensation of any precursors. Taking Al_2_O_3_ ALD as an example, TMA was used as the Al precursor and DI water as the other reactant. TMA was kept at 25 °C to achieve a reasonable vapor pressure. The obtained TMA vapor was carried by ultrahigh purity N_2_ to the reactor. Then, the system was held for several seconds. After that, unreacted precursors and any by-products were removed by ultrahigh purity N_2_ during the reaction. The timing sequence for a typical Al_2_O_3_ ALD was 5 s, 10 s, 180 s, 30 s, 3 s, 10 s, 180 s, and 30 s for TMA dose, system hold, N_2_ flush, evacuation, DI water dose, system hold, N_2_ flush, and evacuation, respectively. ZrO_2_ and HfO_2_ ALD followed a similar procedure. The temperature for the TDMAZ/TDEAH bubbler was held at 80 °C. The time sequence for ZrO_2_ and HfO_2_ ALD was 10 s, 20 s, 240 s, 30 s, 5 s, 20 s, 240 s, and 30 s for TDMAZ/TDEAH dose, system hold, N_2_ flush, evacuation, 30 wt.% hydrogen peroxide dose, system hold, N_2_ flush, and evacuation, respectively. In the ALD process, the number of cycles was chosen so as to obtain the same thickness values for all oxides, i.e., 5 nm for the lower number of cycles and 10 nm for the highest ones. This choice of metal oxide was based on our previous studies [33]. The number of cycles was determined based on different theoretical growth rates. For example, Al_2_O_3_ exhibited a growth rate of 1.3Å per cycle [45], while ZrO_2_ was 1.2Å per cycle [46], slightly lower than that of Al_2_O_3_. HfO_2_ had a growth rate of 0.94 Å per cycle [47].

### 2.2. Coating Characterisation

A Dektak 150 surface profilometer (Bruker, Billerica, MA, USA) with a 2.5 µm stylus diameter was used to measure the Ti(N,O) coatings and the surface roughness of the SS supports as well as all of the deposited coatings. The thickness of the deposited coating was obtained using the edge separating the coating and the uncoated part of the Si coupons. Five lines perpendicular to the edge were measured on each Si coupon. The measured thickness values were averaged, resulting a thickness of about 1 µm (1008 ± 12 nm). All roughness measurements were taken at 10 randomly chosen areas, with the results being averaged. The roughness of each coating was determined over 1 cm during 200 s. Three roughness parameters were used for roughness evaluation and their impact on corrosion resistance: Ra (arithmetic average), Rq (root-mean-square), and Sk (symmetry of the profile about the mean line).

A Hitachi TM3030 Plus (Tokyo, Japan) scanning electron microscope (SEM) coupled to an energy-dispersive X-ray spectrometer (EDS) (Bruker, Billerica, MA, USA) provided the surface morphology and elemental composition of the coatings. The elemental composition measurements were taken at three different areas on each deposited coating, the results were averaged, and the standard deviation (SD) was calculated. The images of surface morphology and elemental composition were obtained by mixed images of backscattering and secondary electrons in one area.

A SmartLab diffractometer (Rigaku, Tokyo, Japan) with CuK_α_ radiation (λ = 0.15405 nm) was used for phase composition investigation. The measurements were performed in a 2θ range of 20° to 100°, using the following parameters: 2° incidence angle, 2°/min. scanning speed, 0.02° step size.

The corrosion process was evaluated by a potentiostat/galvanostat VersaSTAT 3 (Princeton Applied Research, Oak Ridge, TN, USA) and the data were recorded using Versa Studio software (version 2.60.6, Princeton Applied Research, Oak Ridge, TN, USA). 100 mL of 0.9% NaCl + 6% H_2_O_2_ (pH = 4) was used as test medium. The tests were carried out at room temperature (22 ± 1 °C). A standard three electrode cell was used, with platinum as the counter electrode (CE), Ag/AgCl (KCl sat. (0.197 V)) as the reference electrode (RE), and the investigated specimens as the working electrode (WE) (mounted in a Teflon holder with 1 cm^2^ exposed area). After immersion, the specimens were monitored for 1 h, while the open circuit potential (E_oc_) was recorded over time. The time evolution of the potential assumed by the surface in the absence of electrical polarisation (E_oc_) defines its ability to either be oxidised or reduced, as the sample’s surface is already subject to degradation, but the corrosive attack is very slow, as happens in natural conditions. The E_oc_ evolution indicates whether the electrochemical system is stable, or at least stable enough thermodynamically, for a perturbation-based experiment, such as the potentiodynamic polarisation experiment. Even if the oxides are resistant to corrosion, we measured the time evolution of E_oc_ because this is the norm for thin and very thin films due to the possible degradation of the deposited oxides, e.g., Jafari et al. [48]. The potentiodynamic tests were carried out afterward and the corrosion potential (E_corr_), corrosion current density (i_corr_), and anodic (β_a_) and cathodic (β_c_) slopes were directly estimated from Tafel plots, which were recorded from −250 to 250 mV vs. E_oc_, at a scanning rate of 1 mV/s. The polarisation resistance (R_p_) was calculated based on the Stern–Geary equation [49], using the i_corr_ values previously determined, and Tafel anodic and cathodic slopes.

The sample designation indicates the support (SS), the first coating Ti(N,O) deposited by reactive CAE, and the second layer of oxide deposited using ALD along with the number of cycles, e.g., SS/Ti(N,O)/Al_2_O_3_-40c.

## 3. Results 

### 3.1. Surface Morphology and Elemental Composition 

Table 1 presents the elemental composition of the samples. The acquisition time for the EDS analysis was chosen according to the film thickness: 1200 s for Ti(N,O) coatings, about 1 µm thick, and 3600 s for the other six oxynitride/oxide coatings.

**Figure 2:**
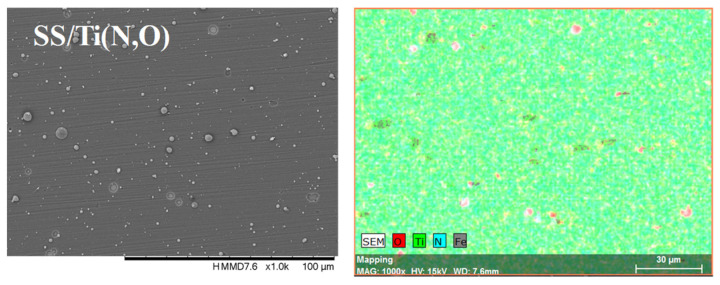
SEM micrographs and composition (EDS) of SS/Ti(N,O) layer.

**Figure 3:**
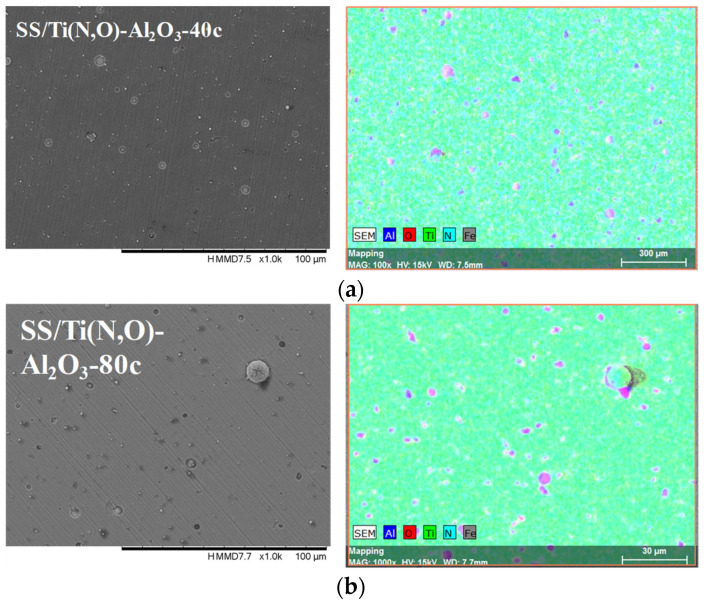
SEM micrographs and composition (EDS) of Al_2_O_3_ layer coated with (**a**) 40 cycles and (**b**) 80 cycles, 1000× magnification.

**Figure 4:**
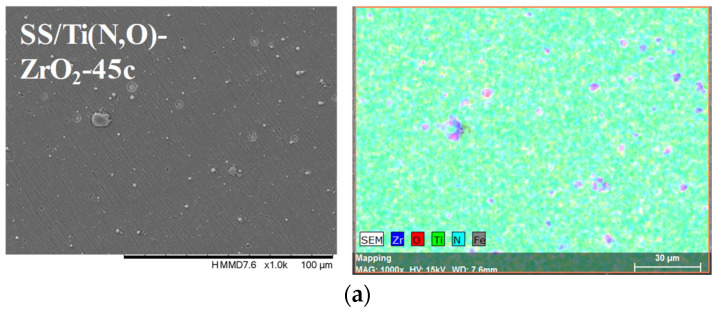

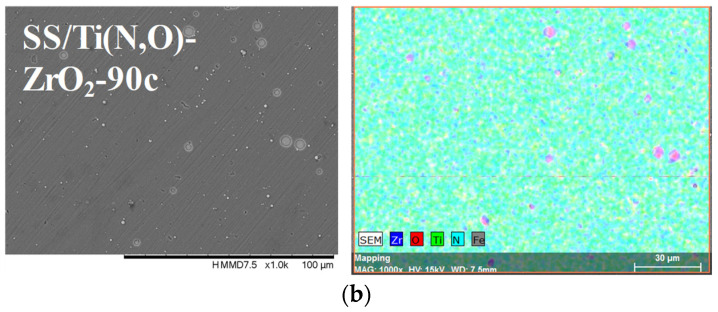
SEM micrographs and composition (EDS) of ZrO_2_ layer coated with (**a**) 45 cycles and (**b**) 90 cycles, 1000× magnification.

**Figure 5:**
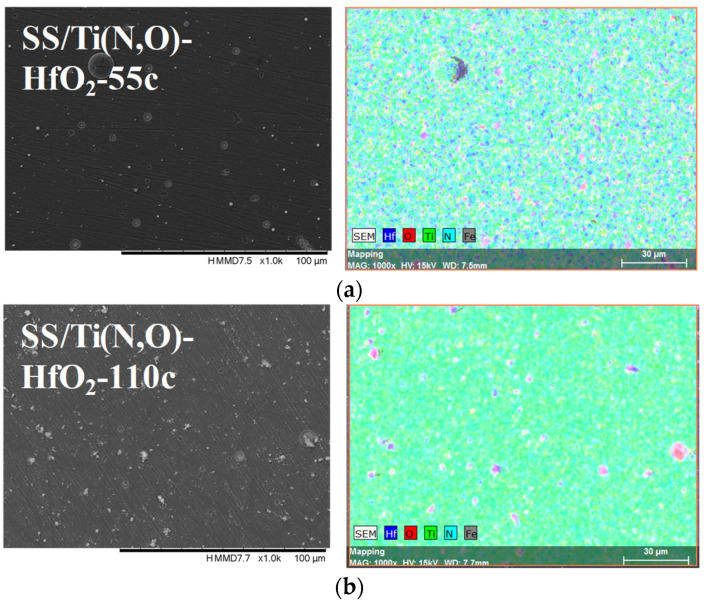
SEM micrographs and composition (EDS) of HfO_2_ layer coated with (**a**) 55 cycles and (**b**) 110 cycles, 1000× magnification.

Figure 2, Figure 3, Figure 4 and Figure 5 show the surface morphology of all coatings. It is noticeable that the few visible pinholes on the SS/Ti(N,O) sample disappeared after ALD coating. The EDS results for each coating, also shown in Figure 2, Figure 3, Figure 4 and Figure 5, indicate the homogeneous distribution of elements on each sample’s surface. The presence of each metal in the ALD-deposited oxides is well visible, including some small metal agglomeration. This comes from the ultrathin nature of ALD. All of the coatings are thin enough that EDS can still clearly detect the metal composition under the coatings. One can observe that the signal corresponding to atomic Fe concentration increased in the case of SS/Ti(N,O)-HfO_2_ coatings, indicating that the bilayers were thinner than the other types of ALD coatings, probably due to the slow growth rate of HfO_2_. The actual thickness of HfO_2_ may be thinner than expected. The designed thickness of HfO_2_ was estimated from the literature. However, the actual growth rate varied because of different conditions such as the dose time and different substrates. The reported growth rate was an average value based on several hundred cycles. The initial growth rate may be slower than the average value. Unlike Al_2_O_3_ and ZrO_2_, HfO_2_ followed an island growth mechanism: nucleation, the development of separated nuclei, and flattening. So, 55 cycles of HfO_2_ may not be enough for a smooth film. Therefore, 110 cycles of HfO_2_ were required to protect the substrate [50]. 

### 3.2. Phase Composition

**Figure 6:**
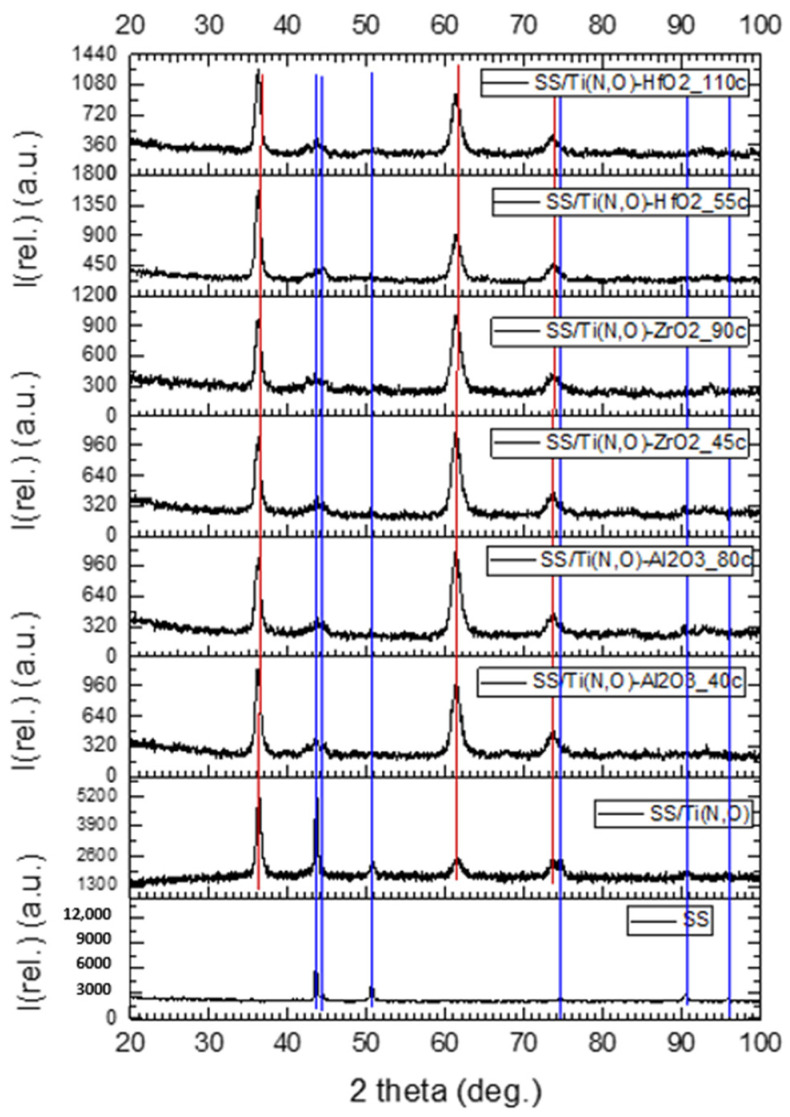
Diffractograms of the 304 L support (SS), the Ti(N,O) coating, and ALD-deposited thin films.

Figure 6 presents the diffractograms of the coatings. The red lines show the maxima specific for the SS and the blue lines those for the Ti(N,O) coatings. Even if the ALD-deposited layer showed no specific signature, it is clear that the ALD process had a certain influence on the SS support, as the support and Ti(N,O) maxima have lower intensity values. The ALD temperature was 200 °C, which is not high enough to crystallise in general. For example, ZrO_2_ starts to crystallise at 420 °C [51]. 

The as-deposited HfO_2_ films were amorphous and remained amorphous after annealing at 400 and 450 °C. At an annealing temperature higher than 500 °C, diffraction peaks appeared, indicating the formation of crystalline HfO_2_ [52]. For a flat support, the amorphous Al_2_O_3_ phase of any thickness was always the most stable in the case of ALD deposition [53,54].

### 3.3. Surface Roughness before and after the Corrosive Attack

**Figure 7:**
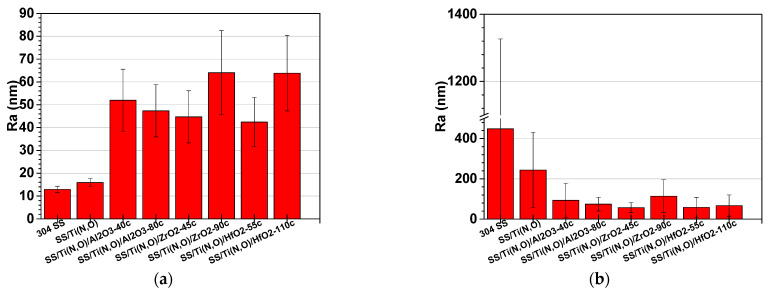
Ra roughness parameter of the samples: (**a**) before and (**b**) after the corrosion tests.

**Figure 8:**
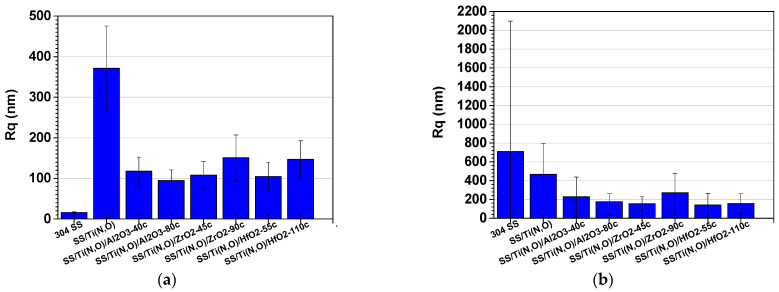
Rq roughness parameter of the samples: (**a**) before and (**b**) after the corrosion tests.

**Figure 9:**
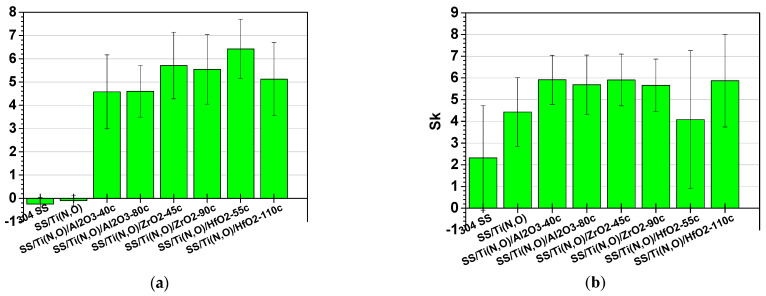
Sk roughness parameter of the samples: (**a**) before and (**b**) after the corrosion tests.

Figure 7, Figure 8 and Figure 9 show the main roughness parameters of the coatings before and after the corrosion attack. The roughness of all samples increased significantly after the corrosion tests. The most significant increase in the Ra and Rq parameters was noted for the SS and SS/Ti(N,O) sample, while in the case of the oxide-coated samples, only a slight increase was observed. Before corrosion, in contrast to the SS and SS/Ti(N,O) samples, the oxide-coated samples exhibited not only higher roughness values, but also a large dispersion of the measured value. 

Due to the quite large non-uniformities present on the surfaces, the roughness parameters of the oxide-coated samples are in the same range, such that the surface roughness could not be further used in the assessment of corrosion resistance. 

**Figure 10:**
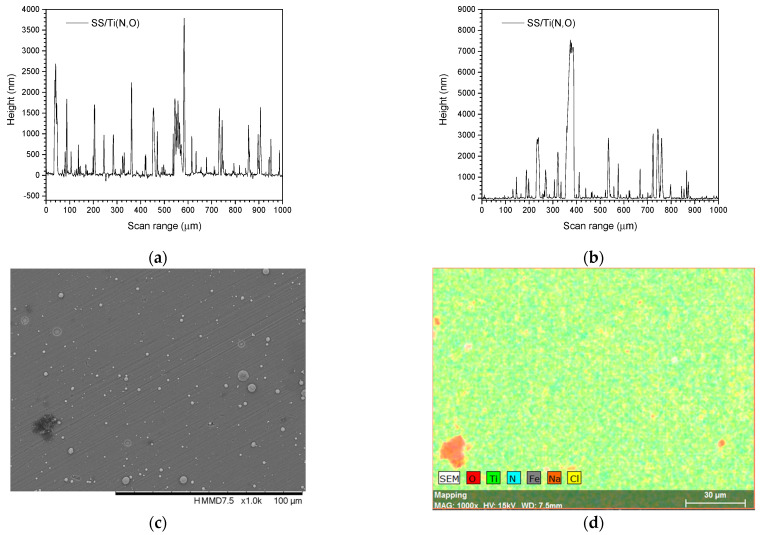
SS/Ti(N,O). Scanned lines obtained by Dektak: (**a**) before and (**b**) after the corrosion test. (**c**) Surface morphology (SEM) and (**d**) elemental composition (EDS) after the corrosion test, magnification 1000×.

**Figure 11:**
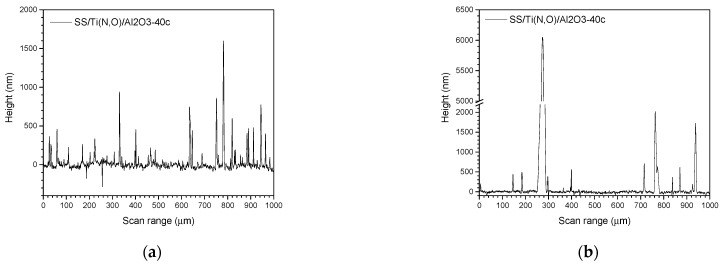

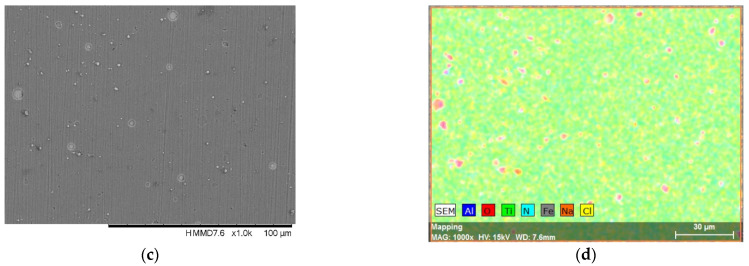
SS/Ti(N,O)/Al_2_O_3_-40c. Scanned lines obtained by Dektak: (**a**) before and (**b**) after the corrosion test. (**c**) Surface morphology (SEM) and (**d**) elemental composition (EDS) after the corrosion test, magnification 1000×.

**Figure 12:**
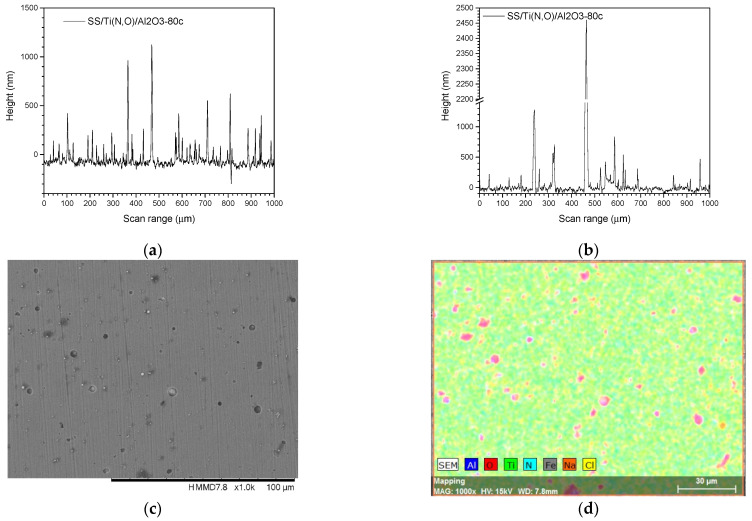
SS/Ti(N,O)/Al_2_O_3_-80c. Scanned lines obtained by Dektak: (**a**) before and (**b**) after the corrosion test. (**c**) Surface morphology (SEM) and (**d**) elemental composition (EDS) after the corrosion test, magnification 1000×.

**Figure 13:**
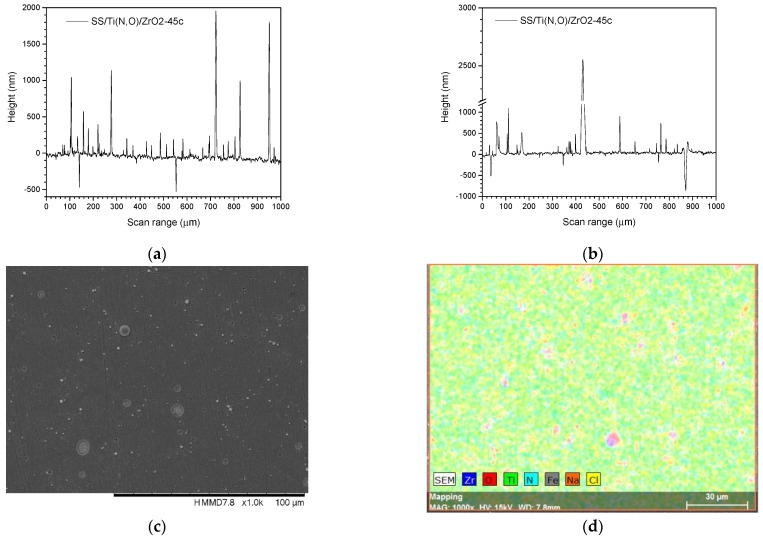
SS/Ti(N,O)/ZrO_2_-45c. Scanned lines obtained by Dektak: (**a**) before and (**b**) after the corrosion test. (**c**) Surface morphology (SEM) and (**d**) elemental composition (EDS) after the corrosion test, magnification 1000×.

**Figure 14:**
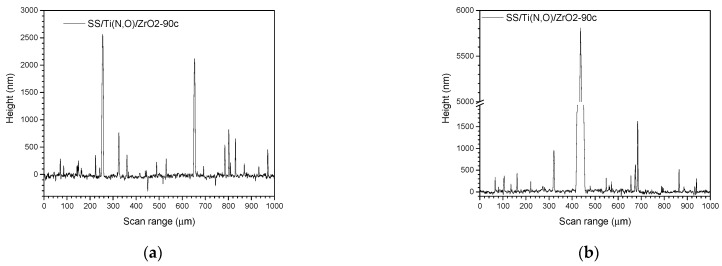

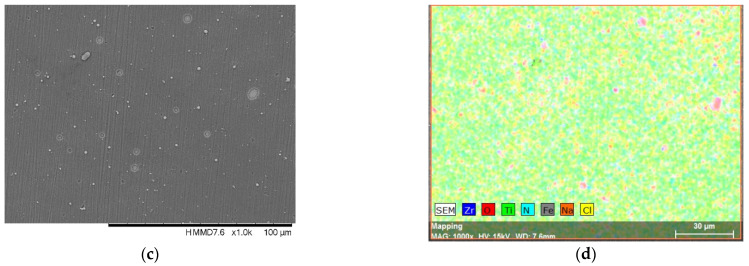
SS/Ti(N,O)/ZrO_2_-90c. Scanned lines obtained by Dektak: (**a**) before and (**b**) after the corrosion test. (**c**) Surface morphology (SEM) and (**d**) elemental composition (EDS) after the corrosion test, magnification 1000×.

**Figure 15:**
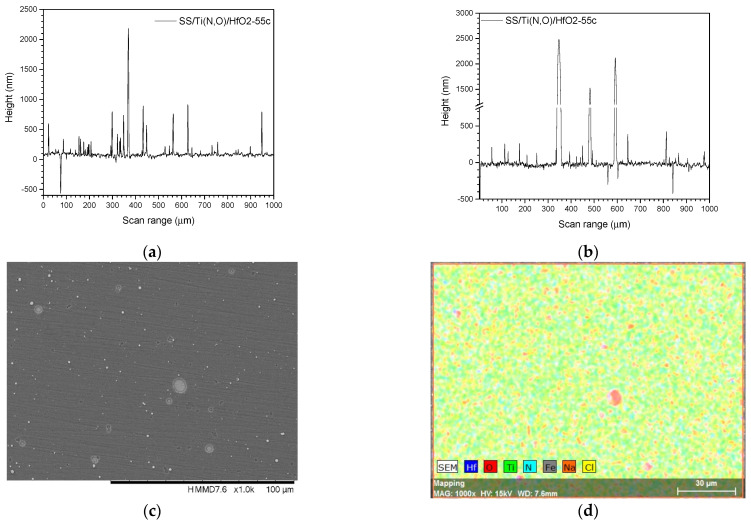
SS/Ti(N,O)/HfO_2_-55c. Scanned lines obtained by Dektak: (**a**) before and (**b**) after the corrosion test. (**c**) Surface morphology (SEM) and (**d**) elemental composition (EDS) after the corrosion test, magnification 1000×.

**Figure 16:**
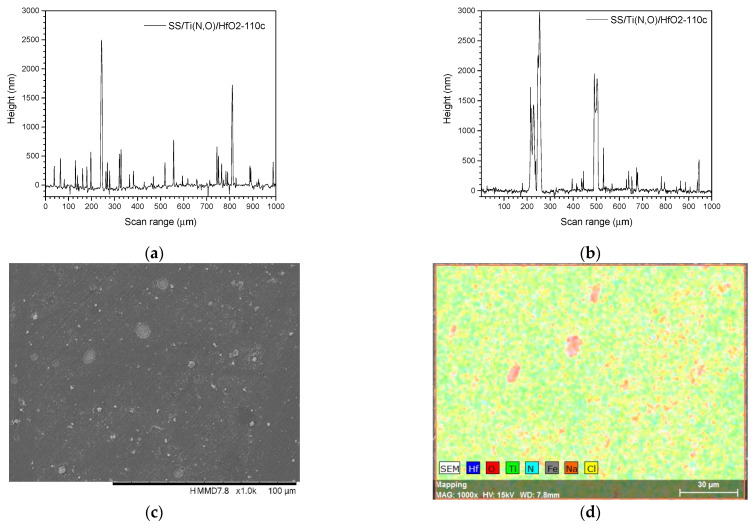
SS/Ti(N,O)/HfO_2_-110c. Scanned lines obtained by Dektak: (**a**) before and (**b**) after the corrosion test. (**c**) Surface morphology (SEM) and (**d**) elemental composition (EDS) after the corrosion test, magnification 1000×.

To sustain this statement, Figure 10, Figure 11, Figure 12, Figure 13, Figure 14, Figure 15 and Figure 16 present the scanned lines obtained by Dektak before and after the corrosion tests showing in detail the surface features responsible for the large error bars, and the morphology (SEM) and composition (EDS) images after the corrosion tests. The corroded surface showed traces of the solution used as electrolyte, as seen in the EDS images, even if the samples were flushed with deionised water after the corrosion test. However, the concentrations (obtained by EDS) of Na and Cl are very low: Cl about 10^−3^ at. % and Na 10^−2^ at.%, and the errors are one order of magnitude lower.

### 3.4. Electrochemical Evaluation—Tafel Plots

**Figure 17:**
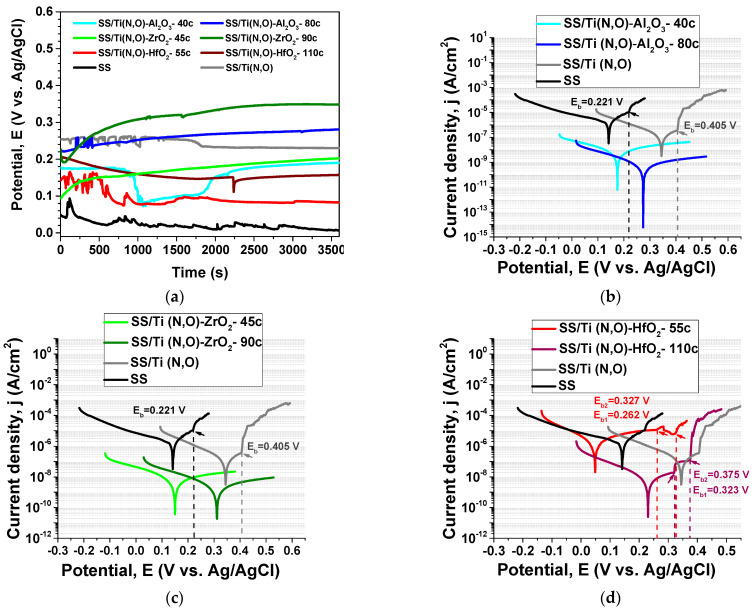
(**a**) Time evolution of E_oc_; Tafel plots of oxides deposited on SS/Ti(N,O): (**b**) Al_2_O_3_, (**c**) ZrO_2_, and (**d**) HfO_2_.

The value of the open circuit potential (E_oc_) depends on the chemical composition of the electrolyte, its temperature and oxygen content, and the nature of the investigated material [55], providing information about the sample’s “nobility”. All coatings presented more electropositive values compared to the SS support (Table 2), indicating a better corrosion resistance due to surface oxidation during the initial immersion in the electrolyte. The time-dependent E_oc_ values measured for all investigated samples are presented in Figure 17a. Even if at E_oc_ the anodic and the cathodic reaction rates are in equilibrium, the increase of E_oc_ during immersion until value stabilisation is usually determined by the decrease in the anodic reaction due to the growth of a passive film [56], and also by the possible increase in the cathodic reaction generated by the increased dissolved oxygen [57]. This trend is observed in the case of thicker alumina and both zirconia-coated samples, indicating that these samples are less prone to corrosion. The highest value was measured for SS/Ti(N,O)-ZrO_2_-90c, followed by SS/Ti(N,O)-Al_2_O_3_-80c. On the other hand, the decrease in E_oc_ indicates an increased susceptibility to corrosion. This is the case for SS, SS/Ti(N,O), and both hafnia-coated samples. 

Considering the Tafel plots (Figure 17b–d), one can see that both the uncoated and Ti(N,O)-coated SS showed a local disruption of the passive layer, evidenced by the breakdown potential (E_b_), giving rise to a sharp increase in the ion current density, indicating that local corrosion processes were activated. E_b_ represents the potential where the current sharply increases with increasing potential, revealing information related to the breakdown of the formed layer during the anodic reactions [57,58]. The E_b_(SS/Ti(N,O) is located at about 405 mV, while E_b_(SS) is located at 221 mV, indicating that, in the bare SS metal, the cathodic process was significantly higher than in the coated one. No breakdown was observed in the case of samples with a top nanolayer of Al_2_O_3_ and ZrO_2_. However, SS/Ti(N,O)/HfO_2_ coatings presented two breakdown values for both thickness values. Additionally, the position of both breakdown potentials related to SS/Ti(N,O)/HfO_2_-55c coating were lower than that related to SS/Ti(N,O). 

The Tafel slopes (βc and βa) were determined from the parts that exhibited linearity in accordance with the Tafel relationship. The calculated values of the cathode and anode slope were all high. However, for the bare SS support, the cathodic slope was significantly higher than the anodic one, indicating a cathodic reduction detrimental to the anodic oxidation [58,59,60]. In the case of SS/Ti(N,O) coatings, the slopes were quite similar, such that it may be concluded that the hydrogen evolution and metal dissolution were almost in equilibrium [61]. For the ALD-coated samples, except for SS/Ti(N,O)/HfO_2_-110c, the anodic slope became higher than the cathodic slope, indicating an inhibition action of the oxides by simply blocking the metal from interaction with the acidic and oxidising environment [62,63,64]. 

The results obtained from the corrosion tests are presented in Table 2.

The corrosion potential E_corr_ can be defined as the potential at which the applied potential changes its polarity; the rate of oxidation is equal to the rate of reduction and is indicative of the kinetic control of the investigated material [57,58]. A high electropositive corrosion potential value (E_corr_) is commonly interpreted to indicate an improved resistance to corrosion. Using this criterion, one can observe that the samples coated with a thicker oxide nanolayer were more resistant to corrosion attack than those with a thinner oxide nanolayer. 

Comparing the corrosion current density values (i_corr_), we observed that the highest value was obtained for SS/Ti(N,O)-HfO_2_-55c. All other coatings exhibited lower i_corr_ values than the bare SS support. This parameter is probably the most important in considering a material’s corrosion resistance. In this respect, the samples coated with a thicker oxide nanolayer were more resistant to corrosion attack than those with a thinner oxide nanolayer, in the following order: SS/Ti(N,O)/Al_2_O_3_-80c > SS/Ti(N,O)/ZrO_2_-90c > SS/Ti(N,O)/HfO_2_-110c. 

The polarisation resistance (R_p_) parameter designates the degree of protection imparted by the passive layer formed on the material’s surface, such that a higher R_p_ value denotes a higher resistance to corrosive attack [65]. The R_p_ parameter has high values for SS/Ti(N,O)/Al2O_3_-80c and SS/Ti(N,O)/ZrO_2_-90c. Both hafnia top-coated samples show low values, in the following order: SS/Ti(N,O)/HfO_2_-55c < SS < SS/Ti(N,O) < SS/Ti(N,O)/HfO_2_-110c < SS/Ti(N,O)/Al_2_O_3_-40c. 

For a comprehensive assessment related to the corrosion resistance of the samples, we applied the Kendall rank correlation [66], considering the parameters with a major influence: E_corr_, i_corr_ and R_p_. Table 3 presents the results for each corrosion parameter labelled from 1 to 8, with rank 1 being related to the best corrosion resistance and rank 8 being assigned to the worst. The last column shows the sum of the ranks (ΣRanks) for each sample, and the best corrosion performance corresponds to the lowest ΣRanks value. Upon summing the ranks, we obtained the same values (4) for SS/Ti(N,O) and SS/Ti(N,O)-ZrO_2_-45c. The last column indicates the coatings’ overall ranks. The coating ordering related to their corrosion resistance may be expressed as: SS/Ti(N,O)/Al_2_O_3_-80c > SS/Ti(N,O)/ZrO_2_-90c > SS/Ti(N,O)/HfO_2_-110c > SS/Ti(N,O)/ZrO_2_-45c = SS/Ti(N,O) > SS/Ti(N,O)/Al_2_O_3_-40c > SS > SS/Ti(N,O)/HfO_2_-55c. The sum of the ranks (ΣRanks) for each sample are calculated such that the highest corrosion performance corresponds to the lowest ΣRanks value. The last column presents the overall rank in which the lower value (1) was ascribed to the best corrosion performance and the highest value (7) to the lowest performance. 

The information derived from the Tafel plots is concurrent with that obtained from the time evolution of E_oc_. Indeed, the SS/Ti(N,O)-ZrO_2_-45c samples was less prone to corrosion compared to SS/Ti(N,O)-Al_2_O_3_-40c, even if the best corrosion resistance was exhibited by the SS/Ti(N,O)-Al_2_O_3_-80c sample. Considering all coated samples, the lowest E_oc_ value was measured for SS/Ti(N,O)-HfO_2_-55c, which presented a lower corrosion resistance, even compared to SS. This result might be explained by the growth mechanism of HfO_2_, such that the thinner hafnia films might not have been continuous.

The results obtained for the thinner oxide coatings indicate that during the preparation of oxide coatings, the use of the hydrogen peroxide started to aggressively corrode the Ti(N,O) and possibly also the SS support. Due to the island mechanism of growth of the hafnia coating, the corrosion attack of the electrolyte in the highly oxidative environment depreciated both the Ti(N,O) coatings and the SS support, resulting in the poor corrosion resistance of the SS/Ti(N,O)/HfO_2_-55c sample. The superior results obtained for SS/Ti(N,O)/ZrO_2_-45c compared to SS/Ti(N,O)/Al_2_O_3_-40c may be ascribed to the higher E_oc_ values obtained for the thin zirconia coating. This result is also concurrent with the fact that the ALD technique is sensitive to the chemical state of the substrate used, and in the case of alumina, its growth is eased if the support surface is rich in hydroxyl groups [67,68]. Due to this peculiarity of ALD-grown alumina, the proper nucleation of film was impeded because the support had insufficient hydroxyl groups, as these were consumed by etching the Ti(N,O) initial layer. We infer that, at the beginning of the growth process, a slight decrease in the sealing properties of the first alumina oxide layers occurred, such that SS/Ti(N,O) outperformed its corrosion resistance. 

The mechanisms responsible for the different electrochemical properties of the thinner oxide coatings might be also related to the very thin layer of oxide and to the possibility of two opposing reactions occurring: the hot hydrogen peroxide partially removing the Ti(N,O) and starting to corrode the SS support, and the common oxidation reaction that forces the oxide layer to form. 

Both the thickness of the coating and the coating materials are important. In this study, thin films with ~10 nm thickness exhibited good corrosion resistance, which was much thinner than most of the reported values due to the high quality of our ALD thin films. For example, ∼50 nm ALD films of Al_2_O_3_ (64 nm), TiO_2_ (40 nm), ZnO (50 nm), ZrO_2_ (60 nm), and HfO_2_ (60 nm) was required for the corrosion protection of copper [32]. A 70 nm TiO_2_ film was used to protect magnesium alloys [69]. The considerably thinner ALD films we prepared were still beneficial to maintain the original function of the primary coated supports.

Summarising, we conclude that the thicker ALD oxide coatings improved the corrosion resistance of the Ti(N,O)-coated 304 L stainless steel. The best results were obtained when using alumina and zirconia as upper coatings. Despite the quite high roughness of the coatings after corrosion, the coatings blocked the attack of the corrosive solution. The less promising results obtained for thin hafnia nanolayer-coated samples might be related to the thinner coating of HfO_2_ than Al_2_O_3_ and ZrO_2_ due to its lower growth rate and specific island growth mechanism, which is consistent with the hafnia composition shown in Table 1, pointing to a certain minimum thickness required for the ALD-deposited oxide. This detail should be further studied as it is likely a specific characteristic of each oxide. This is an example of what happens in an advanced oxidation system and explains why the best ALD coating selection is needed to achieve the most corrosion-resistant material coatings. This study neatly shows that the standard anticorrosion coatings require significant improvement for applications such as advanced oxidation. Moreover, it shows which coatings are likely to be robust enough to protect SS in such highly oxidative environments, and which do not offer any advantage. 

## 4. Conclusions

We report on the deposition on stainless steel supports of two different layers with different compositions, Ti(N,O) oxynitride, obtained using CAE, and alumina, zirconia, or hafnia deposited using ALD. Though Ti(N,O)-coated 304L stainless steel proved to be quite corrosion resistant in the applied acidic, saline, and oxidising environment, the presence of some pinholes specific for the cathodic arc evaporation still renders it vulnerable to oxidation and corrosion. This work aimed to increase the protection of the surface of Ti(N,O)-coated 304L stainless steel in an aqueous acidic, saline, oxidising solution by nanometre ultrathin Al_2_O_3_, ZrO_2_, and HfO_2_ ALD coatings. The corrosion protection behaviour of the fabricated coatings was assessed by potentiodynamic polarisation tests in a simulated corrosive environment. The effect of the number of ALD cycles on ceramic film thickness and corrosion resistance was also investigated. Two thickness values of the oxides were deposited. The surfaces coated by oxynitride and oxides presented a good corrosion resistance, with the best results being obtained for the thicker oxides, which outperformed the corrosion resistance of Ti(N,O)-coated 304L stainless steel. The thicker Al_2_O_3_, ZrO_2_, and HfO_2_ ALD coatings with ~10nm thickness showed excellent corrosion resistance, while the thinner Al_2_O_3_, ZrO_2_, and HfO_2_ ALD coatings exhibited different performances. Thinner HfO_2_ films had lower corrosion resistance than Al_2_O_3_ and ZrO_2_ films. The reason for this may be due to the different growth mechanisms. The island growth mechanism of HfO_2_ required more cycles of coating to efficiently protect the substrate. The thinner oxide coatings had a lower corrosion resistance, with the lowest performance being observed for the thin hafnia coating SS/Ti(N,O)/HfO_2_-55c. Due to the conformal nature of ALD coatings, the corrosion of droplets specific for the coatings obtained using cathodic arc evaporation could be efficiently contained, even with a thickness of only several nanometres, blocking the metal from interactions with the acidic and oxidising environment. 

The obtained results indicated that the use of CAE and ALD deposition methods can obtain coatings with higher protection resistance to corrosive attack in saline and acidic environments.

## Figures and Tables

**Table 1:** Elemental composition of the investigated specimens.

Sample	Al (at.%)	Zr (at.%)	Hf (at.%)	O (at.%)	Ti (at.%)	N (at.%)	Fe (at.%)
**SS/Ti(N,O)**	-	-	-	8.95 ± 0.53	52.60 ± 2.28	47.58 ± 1.72	0.87 ± 0.07
**SS/Ti(N,O)-Al_2_O_3_-40c**	1.802 ± 0.106			28.13 ± 1.72	41.61 ± 2.09	27.36 ± 1.44	1.09 ± 0.09
**SS/Ti(N,O)-Al_2_O_3_-80c**	2.887 ± 0.165			37.29 ± 2.43	37.11 ± 2.25	21.82 ± 1.25	0.89 ± 0.082
**SS/Ti(N,O)-ZrO_2_-45c**		0.710 ± 0.102		23.18 ± 1.28	48.06 ± 2.14	26.77 ± 1.26	1.288 ± 0.09
**SS/Ti(N,O)-ZrO_2_-90c**		1.832 ± 0.230		37.70 ± 2.10	44.28 ± 2.49	15.05 ± 0.75	1.14 ± 0.09
**SS/Ti(N,O)-HfO_2_-55c**			0.006 ± 0.001	8.05 ± 0.49	50.85 ± 2.18	39.70 ± 1.80	1.40 ± 0.10
**SS/Ti(N,O)-HfO_2_-110c**			0.540 ± 0.127	17.06 ± 1.01	47.99 ± 2.17	33.06 ± 1.60	1.36 ± 0.10

**Table 2:** Corrosion parameters (open circuit potential—E_oc_, corrosion potential—E_corr_, corrosion current density—i_corr_, polarisation resistance—R_p_).

Sample	E_oc_ (mV)	E_corr_ (mV)	i_corr_ (nA/cm^2^)	βc (mV)	βa (mV)	R_p_ (kΩ)
**SS**	6	142	1362	182.98	67.75	16
**SS/Ti(N,O)**	230	345	174	144.961	132.523	173
**SS/Ti(N,O)-Al_2_O_3_-40c**	190	171	12.6	240	509	5627
**SS/Ti(N,O)-Al_2_O_3_-80c**	281	271	0.5	142	287	82,646
**SS/Ti(N,O)-ZrO_2_-45c**	202	152	10.3	220	613	6846
**SS/Ti(N,O)-ZrO_2_-90c**	348	311	4.5	231	654	16,503
**SS/Ti(N,O)-HfO_2_-55c**	83	48	4344	185	428	13
**SS/Ti(N,O)-HfO_2_-110c**	157	231	7.7	127	130	3627

**Table 3:** Kendall ranks attributed to the coated samples according to three corrosion parameters: corrosion potential—E_corr_, corrosion current density—i_corr_, polarisation resistance—R_p_.

Sample	Rank-E_corr_	Rank-i_corr_	Rank-R_p_	ΣRanks	Overall Rank
**SS**	7	7	7	21	6
**SS/Ti(N,O)**	1	6	6	13	4
**SS/Ti(N,O)-Al_2_O_3_-40c**	5	5	4	14	5
**SS/Ti(N,O)-Al_2_O_3_-80c**	3	1	1	5	1
**SS/Ti(N,O)-ZrO_2_-45c**	6	4	3	13	4
**SS/Ti(N,O)-ZrO_2_-90c**	2	2	2	6	2
**SS/Ti(N,O)-HfO_2_-55c**	8	8	8	24	7
**SS/Ti(N,O)-HfO_2_-110c**	4	3	5	12	3

## Data Availability

https://www.mdpi.com/ethics (accessed on 30 December 2022).
